# Which Method for Diagnosing Small Fiber Neuropathy?

**DOI:** 10.3389/fneur.2020.00342

**Published:** 2020-05-05

**Authors:** Vincent Fabry, Angélique Gerdelat, Blandine Acket, Pascal Cintas, Vanessa Rousseau, Emmanuelle Uro-Coste, Solène M. Evrard, Anne Pavy-Le Traon

**Affiliations:** ^1^Department of Neurology, Toulouse University Hospital, Toulouse, France; ^2^University of Toulouse III Paul Sabatier, Toulouse, France; ^3^Neurology, Clinique des Cèdres, Toulouse, France; ^4^MeDatAS Unit, Department of Medical and Clinical Pharmacology, Toulouse University Hospital, Toulouse, France; ^5^Department of Pathology, Toulouse University Hospital, IUC-Oncopole, Toulouse, France; ^6^INSERM U1037, Cancer Research Center of Toulouse (CRCT), Toulouse, France; ^7^Institute of Cardiovascular and Metabolic Diseases (I2MCUMR1048), Toulouse, France

**Keywords:** Small fiber neuropathy (SFN), pain, autonomic nervous system, skin biopsy, neurophysiology

## Abstract

**Introduction:** Small fiber neuropathies (SFN) induce pain and/or autonomic symptoms. The diagnosis of SFN poses a challenge because the role of skin biopsy as a reference method and of each neurophysiological test remain to be discussed. This study compares six methods evaluating small sensory and autonomic nerve fibers: skin biopsy, Quantitative Sensory Testing (QST), quantitative sweat measurement system (Q-Sweat), Laser Evoked Potentials (LEP), Electrochemical Skin Conductance (ESC) measurement and Autonomic CardioVascular Tests (ACVT).

**Methods:** This is a single center, retrospective study including patients tested for symptoms compatible with SFN between 2013 and 2016 using the afore-mentioned tests. Patients were ultimately classified according to the results and clinical features as “definite SFN,” “possible SFN” or “no SFN.” The sensitivity (Se) and specificity (Sp) of each test were calculated based on the final diagnosis and the best diagnostic strategy was then evaluated.

**Results:** Two hundred and forty-five patients were enrolled (164 females (66.9%), age: 50.4 ± 15 years). The results are as follows: skin biopsy: Se = 58%, Sp = 91%; QST: Se = 72%, Sp = 39%; Q-Sweat: Se = 53%, Sp = 69%; LEP: Se = 66%, Sp = 89%; ESC: Se = 60%, Sp = 89%; Cardiovascular tests: Se = 15%, Sp = 99%. The combination of skin biopsy, LEP, QST and ESC has a Se of 90% and a Sp of 87%.

**Conclusion:** Our study outlines the benefits of combining skin biopsy, ESC, LEP and QST in the diagnosis of SFN.

## Introduction

Small fiber neuropathies (SFN) are peripheral neuropathies involving small and thinly myelinated fibers (Ad) and unmyelinated (C) nerve fibers. Large fibers may be unaffected (pure SFN) or affected (mixed neuropathy). Patients with SFN usually present with neuropathic pain, paraesthesia, dysesthesia and/or thermo-algic hypoesthesia, but can also experience autonomic symptoms (orthostatic hypotension, urinary or digestive disorders, dry eye or mouth syndrome). The topography of the sensitive symptoms may or may not be length-dependent (1). Thus, the clinical signs of SFN are not very specific.

Electrophysiological diagnosis of SFN poses a challenge for neurologists. Indeed, nerve conduction studies are unaffected in terms of pure SFN. Therefore, several specific diagnostic methods have been studied exploring either sensitive or autonomic fibers: Intra-Epidermal Nerve Fiber Density (IENFD) evaluation by skin biopsy, (2–4) Quantitative Sensory Testing (QST), (5, 6) quantitative sweat measurement system (Q-Sweat), (7, 8) Laser Evoked Potentials (LEP), (9–11) and recently Electrochemical Skin Conductance (ESC) measurement (12–14, 14) (Table 1). We also performed Autonomic CardioVascular Testing (ACVT) to assess the autonomic nervous system.

**Table 1:** Description of the methods.

**Number of sensory fibers**	**Functional evaluation of small fibers**				
**Skin biopsy**	**QST**	**LEP**	**Q-Sweat**	**ESC**	**ACVT**
Intra-epidermal nerve fiber density	Sensory fibers		Autonomic C fibers		
	C	Aδ			

*The table summarizes the different methods and the type of fiber analyzed*.

*QST, Quantitative Sensory Testing; LEP, Laser Evoked Potentials; ESC, Electrochemical Skin Conductance measurement; ACVT, Autonomic Cardio-Vascular Tests*.

However, data comparing the diagnostic value of these methods for the diagnosis of SFN are scarce, and there is currently no consensus on the type and number of tests to be performed. While some authors have suggested that two abnormal tests are necessary to confirm the diagnosis of SFN, these criteria take into account only a few tests (skin biopsy and QST in the very first criteria published by Devigili et al., skin biopsy, QST and Q-Sweat for Thaisethawattkul et al.). Tesfaye et al. proposed criteria for diabetic SFN (NEURODIAB criteria) with different levels of probability including probable SFN (with clinical criteria associating length-dependant symptoms, clinical signs of small fiber and normality of nerve conduction on the sural nerve) and definite SNF (requiring an abnormal IENFD and/or an abnormal QST at the foot) (15).

The purpose of this study was to determine the diagnostic value of skin biopsy, QST, Q-Sweat, LEP, ESC measurement and cardio-vascular testing for the diagnosis of SFN, and then to evaluate the most relevant diagnostic strategy.

## Methods

### Patient Population

Two hundred and forty-five patients referred to the laboratory with suspected SFN were enrolled in the study. Cinical signs (such as pinprick and thermal sensory loss, allodynia or hypoesthesia) were not mandatory inclusion.

The cohort comprised 164 (66.9%) women from 11 to 85 years of age (mean: 50.4 ± 15.0).

These patients underwent small fiber evaluation between August 2013 and January 2016 with the following inclusion criteria: (1) sensory and/or autonomic symptoms consistent with SFN; (2) normal nerve conduction (normal ulnar, median, sural, superficial peroneal sensory responses and ulnar, median, tibial, peroneal motor responses with F-waves) according to our laboratory normative values.

If the clinical examination was consistent with an involvement of the central nervous system or if only LEP or QST were abnormal, we performed an MRI of the central nervous system so as not to miss a brain or spinal cord injury.

The French National Commission for Data Protection (*CNIL*) was notified before this single-center (Toulouse University Hospital, Toulouse, France), retrospective cohort study was carried out using retrospective data gleaned from hospital medical records.

### Description of Tests

All of the tests were performed in succession over 1 day: QST, Q-Sweat, LEP, CVT, ESC measurement and skin biopsy.

QST was performed on the hand and foot (on the right side, unless symptoms were lateralised only on the left side of the body) using Thermotest® device (Somedic, Sollentuna, Sweden) and the “method of limits” as described by Rolke et al. (16). We set the warm detection threshold and heat pain threshold as the absolute difference between the measured threshold (average of three trials for each threshold) and the baseline temperature of 32°C. Threshold values were analyzed with normative values established by Rolke et al. (17).

Q-Sweat was performed in 3 sites: foot, proximal leg and forearm (on the right side, unless symptoms were lateralised on the left side of the body) using the Q-SWEAT® device (WR Medical Electronic, Minneapolis, USA). (18) Sweat volume was recorded for each site. The results were analyzed in comparison with normative values published by Novak (18).

LEPs were recorded using a YAG laser (Medtronic, Dublin, Ireland; wavelength: 1340 nm, power: 0.5 to 15 J, beam diameter: 4 mm) coupled to a Medelec Synergy® device (Oxford Instruments, Abingdon, UK) and a scalp EEG electrode placed at Cz with reference to earlobes. The laser stimulation was performed on the dorsum of the hand and feet (on the right side, unless symptoms were lateralised on the left side of the body). The first step was a “psychophysical” approach allowing the operator to determine both sensitive and painful thresholds. LEPs were then recorded using an intensity corresponding to a painful threshold. Amplitudes and latencies were measured and compared to normative values published by Devos et al. (19).

ACVT was performed using continuous ECG and blood pressure recordings by digital photoplethysmography (NEXFIN®, BMEYE B.V., Amsterdam, Netherlands). ACVT included 5 tests (“Ewing tests”) that generate an autonomic neuropathy score (“Ewing score” ranging from 0 to 5). These procedures investigate study variations in heart rate and/or blood pressure with Valsalva maneuver, deep breathing, standing up, and sustained handgrip, as described by Ewing et al. (20) The variations recorded during the cardiovascular tests were interpreted using the normative values of our laboratory, which are in accordance with the values reported by Low and Benarroch (21).

ESC was measured using the Sudoscan® device (Impeto Medical, Paris, France). The hands and feet of each patient were placed for 2 min on stainless steel metal electrodes to which a low-voltage electrical current was applied. The ESC was automatically calculated using the device for palms and soles (expressed in μS). The normative values of skin conductance used in our center are those recommended by the manufacturer: skin conductance is considered normal if it exceeds 70 and 60 μS in the feet and hands, respectively.

A skin biopsy was performed with a 3-mm punch in a single site in the distal leg and processed as described by Lauria et al. (2, 3) in order to determine the IENFD. IENFD values were compared to normative values published by Lauria et al. (2) the biopsy was considered abnormal if the IEFND was lower than anticipated for age and gender (<5^th^ percentile).

### Final Diagnosis

By analogy with published criteria, (22, 23) we used anomalies in at least 2 of the 6 tests as diagnostic criteria for SFN.

At the end of the evaluation, two neurologists summarized all of the clinical data and the test results. Each patient received a diagnosis: “definite SFN” if the criteria were met, “no SFN” if the criteria were not met. In certain situations (limit values, results possibly modified by treatments or pre-existing condition, abnormalities inconsistent with the symptoms, a single abnormal test but highly suggestive clinical picture), the diagnosis of SFN was considered “possible.”

For patients with a final diagnosis of “definite SFN,” the most probable cause was documented (if data were available).

### Statistical Analysis

Descriptive analyses were performed including frequency and percentage for qualitative characteristics and mean, standard deviation (SD), minimum and maximum for quantitative characteristics. Population characteristics and the results of the investigations were compared between the three groups (“Definite SFN,” “No SFN” and “Possible SFN”) using the Chi2 (Fisher Test as appropriate) for qualitative variables and Wilcoxon's rank sum test for quantitative variables.

The diagnostic performances of the tests (skin biopsy, QST, Q-Sweat, ESC measurement, LEP, and ACVT) were studied by sensitivity/specificity analysis. For the analyses, the results of these 5 tests were considered either “Normal” or “Abnormal,” and the reference was the final diagnosis of SFN which comprised 2 groups: “Definite SFN” and “No SFN.” Sensitivity was therefore the ability of a test to correctly classify a patient as “Definite SFN” and specificity was the ability of a test to correctly classify a patient without SFN (“No SFN”). The Positive Predictive Value (PPV) and Negative Predictive Value (NPV) were calculated. PPV was the percentage of patients with a normal test and final diagnosis of “Definite SFN” and NPV was the percentage of patients with an abnormal test and a final diagnosis of “No SFN.”

Finally, the aim was to determine the best strategy for the diagnosis of SFN: after selecting the 4 tests with the best sensitivity/specificity outcomes, we calculated sensitivity, specificity, PPV and NPV for each possible combination of these 4 tests (always using the abnormality of two tests as the SFN criterion).

All *P-*values were two-sided and only *P*-values below 0.0001 were considered statistically significant in order to limit the risk of false positive results. SAS® statistics software, version 9.4 (SAS Institute Inc., Cary, NC) was used for all analyses.

## Results

### Description of the Entire Cohort

Patients' characteristics are shown in Table 2 In terms of symptoms, patients were mainly referred for pain (191 patients, 79.9%). Of the 212 patients for whom symptom topography was documented, the findings were length-dependant in 54.7% of cases. The mean DN4 score was 4.7 ± 2.0.

**Table 2:** Population characteristics.

					**Group comparison**		
	**Total population**	**Definite SFN**	**Possible SFN**	**No SFN**	**“Definite SFN” and “No SFN”**	**“Definite SFN” and “Possible SFN”**	**“No SFN” and “Possible SFN”**
	*n* = 245	*n* = 102	*n* = 53	*n* = 90			
Age (years)	50.4 ± 15.0 (11–85)	55.1 ± 15.5 (11–85)	46.7 ± 13.6 (21–71)	47.1 ± 14.0 (18–76)	0.0003	0.001	0.85
Gender							
Women	164 (66.9%)	61 (59.8%)	35 (66.0%)	68 (75.6%)	0.02	0.45	0.22
Men	81 (33.1%)	41 (40.2%)	18 (34.0%)	22 (24.4%)			
BMI (kg/m^2^)	24.2 ± 4.8 (15.6–40.4)	25.1 ± 5.1 (15.6–40.4)	23.9 ± 5.3 (16–38.6)	23.4 ± 4.1 (16.8–35.3)	0.01	0.15	0.62
Age of symptoms	*n* = 170	*n* = 73	*n* = 36	*n* = 61			
<1 year	29 (17.1%)	12 (16.4%)	8 (22.2%)	10 (16.4%)	0.18		
1–5 years	93 (54.7%)	36 (49.3%)	19 (52.8%)	38 (62.3%)		0.45	0.64
> 5 years	48 (28.2%)	25 (34.3%)	9 (25.0%)	13 (21.3%)			
Symptoms	*n* = 239	*n* = 102	*n* = 49	*n* = 88			
Pain	191 (79.9%)	88 (86.3%)	439 (79.6%)	64 (72.3%)	0.10^*^	0.11^*^	0.10^*^
Paraesthesia	19 (7.9 %)	8 (6.9%)	1 (2.0%)	11 (12.5%)			
Autonomic symptoms	26 (10.9%)	5 (4.9%)	8 (16.3%)	13 (14.8%)			
Others (restless legs, etc.)	3 (1.3%)	2 (2.0%)	1 (2.0%)	0 (0.0%)			
Topography of symptoms	*n* = 212	*n* = 94	*n* = 44	*n* = 74			
Length-dependent	116 (54.7%)	55 (58.5%)	27 (61.4%)	34 (46.0%)	0.11	0.75	0.11
Non-length-dependent	96 (45.3%)	39 (41.5%)	17 (38.6%)	40 (54.0%)			
DN4 score	*n* = 177	*n* = 74	*n* = 37	*n* = 66			
	4.7 ± 2.0 (0–8)	4.8 ±2.1 (0–8)	4.8 ± 2.0 (0–8)	4.6 ± 2.1 (0–8)	0.40	0.81	0.61
Taking at least one drug that may change the test results	*n* = 210	*n* = 90	*n* = 45	*n* = 75			
	59 (28.1%)	26 (28.9%)	16 (35.6%)	17 (22.7%)	0.36	0.43	0.13

*SFN, Small Fiber Neuropathy; BMI, Body Mass Index. Wilcoxon tests are performed for the comparisons of quantitative variables, and Chi2 tests for qualitative variables*.

* *Fisher test*.

### Comparison of the “No SFN” and “Definite SFN” Groups

Characteristics of the three groups are compared in Table 2. Although there was no significant difference between groups in terms of weight, age or gender, patients in the “definite SFN” group tended to be older with a higher BMI than those in the “no SFN” group (55.1 years vs. 47.1, *P* = 0.0003; 24.2 kg/m2 vs. 23.4, *P* = 0.01). The DN4 score did not differ between the “definite SFN” and “No SFN” groups (4.8 vs. 4.6, *P* = 0.40).

### Causes of SFN

**Figure 1:**
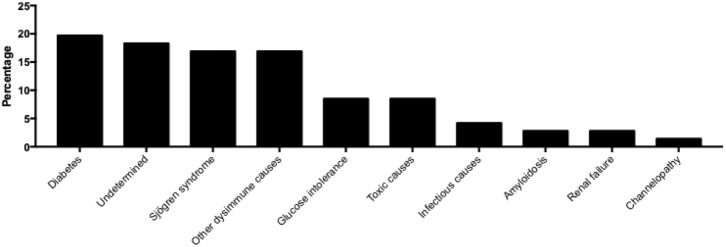
Prevalence of possible cause in patients with definite Small Fiber Neuropathy for whom aetiological investigations were documented.

The most probable causes have been documented for 71 patients with definite SFN and summarized in Figure 1. The most frequent causes are diabetes (14 patients, 19.7%), associated with glucose intolerance (6 patients, 8.5%), followed by Sjögren's syndrome (12 patients, 16.9%) and other dysimmune causes (12 patients, 16.9%).

### Comparison of the Results of the Explorations Based on the Final Diagnosis

The results of each exploration based on the final diagnosis (“Definite SFN,” “Possible SFN” or “No SFN”) are compared in Table 3.

**Table 3:** Exploration results (based on the final diagnosis).

				**Group comparison**			
	**Total population**	**Definite SFN**	**Possible SFN**	**No SFN**	**“Definite SFN” and “No SFN”**	**“Definite SFN” and “Possible SFN”**	**“No SFN” and “Possible SFN”**
	*n* = 245	*n* = 102	*n* = 53	*n* = 90			
**Skin biopsy**							
IntraEpidermal nerve Fiber Density (fibers/mm)	6.21 ± 3.41	4.61 ± 3.04	6.58 ± 4.01	7.83 ± 2.53	**<0.0001**	0.0006	0.003
**Quantitative Sensory Testing (QST)**							
Perceptual threshold in the foot (°C)	43.5 ± 4.25	44.48 ± 4.13	43.11 ± 4.18	42.62 ± 4.24	0.003	0.05	0.44
Heat painful threshold in the foot (°C)	46.94 ± 3.12	47.32 ± 3.2	47.06 ± 2.49	46.43 ± 3.31	0.03	0.08	0.52
Perceptual threshold in the hand (°C)	37.97 ± 4.09	38.84 ± 4.39	37.35 ± 3.38	37.34 ± 3.97	0.004	0.06	0.28
Heat pain threshold in the hand (°C)	45.09 ± 4.33	44.97 ± 4.74	45.4 ± 3.99	45.06 ± 4.04	0.88	0.87	0.72
**Quantitative sweat measurement (Q-Sweat)**							
Sweat volume (foot) (μL)	0.32 ± 0.31	0.28 ± 0.28	0.35 ± 0.42	0.34 ± 0.28	0.05	0.45	0.31
Sweat volume (proximal leg) (μL)	0.45 ± 0.42	0.41 ± 0.44	0.5 ± 0.48	0.47 ± 0.36	0.02	0.15	0.61
Sweat volume (forearm) (μL)	0.39 ± 0.45	0.39 ± 0.53	0.39 ± 0.38	0.38 ± 0.4	0.18	0.30	0.99
**Electrochemical Skin Conductance (ESC) study**							
Mean conductance in the hands (μS)	66.36 ± 15.62	60.09 ± 16.87	64.32 ± 15.95	74.67 ± 8.95	**<0.0001**	0.20	0.0004
Mean conductance in the feet (μS)	75.73 ± 13.6	70.08 ± 16.63	76.71 ± 11.26	81.56 ± 6.88	**<0.0001**	0.02	0.002
**Laser Evoked Potentials (LEP)**							
N2P2 complex amplitude (foot stimulation) (μV)	18.78 ± 13.78	13.94 ± 13.41	19.68 ± 14.04	23.98 ± 12.13	**<0.0001**	0.01	0.10
N2 latency (foot stimulation) (ms)	287.16 ± 56.48	308.27 ± 63.72	287.35 ± 64.93	269.29 ± 36.14	0.0005	0.06	0.33
P2 latency (foot stimulation) (ms)	398.13 ± 66.03	420.88 ± 70.7	401.85 ± 71.27	377.01 ± 51.62	0.0008	0.10	0.21
N2P2 complex amplitude (hand stimulation) (μV)	21.79 ± 16.09	18.48 ± 18.03	22.38 ± 15.29	25.45 ± 13.14	0.0003	0.07	0.22
N2 latency (hand stimulation) (ms)	221.85 ± 41.78	232.41 ± 50.16	215.5 ± 29.51	215.32 ± 37.02	0.01	0.15	0.51
P2 latency (hand stimulation) (ms)	320.27 ± 54.73	326.83 ± 66.96	317.81 ± 42.72	315.38 ± 47.3	0.52	0.79	0.62
**Cardio-vascular Testing (CVT)**							
Baseline systolic blood pressure (mmHg)	124.45 ± 15.65	127.68 ± 16.36	120.64 ± 15.49	123.02 ± 14.31	0.01	0.001	0.21
Baseline diastolic blood pressure (mmHg)	73.46 ± 9.62	73.42 ± 10.16	72.83 ± 9.56	73.9 ± 9.08	0.83	0.86	0.71
Valsalva ratio	1.67 ± 1.06	1.72 ± 1.6	1.6 ± 0.25	1.65 ± 0.33	0.06	0.22	0.56
Heart rate variation during deep breathing (bpm)	17.98 ± 8.57	16.32 ± 8.96	18.67 ± 7.53	19.4 ± 8.47	0.01	0.03	0.74
30:15 ratio	1.27 ± 0.19	1.23 ± 0.18	1.26 ± 0.16	1.31 ± 0.21	0.005	0.14	0.29
SBP during isometric handgrip (mmHg)	24.05 ± 15.17	23.4 ± 14.47	24.66 ± 17.97	24.45 ± 14.32	0.73	0.93	0.66
DBP variation during isometric handgrip (mmHg)	16.30 ± 8.95	16.86 ± 9.44	15.00 ± 9.10	16.41 ± 6.87	0.83	0.27	0.16
SBP variation in upright posture (mmHg)	−3.42 ± 13.72	−3.75 ± 14.46	−5.38 ± 14.58	−1.89 ± 12.25	0.49	0.75	0.37
DB variation in upright posture (mmHg)	3.31 ± 8.95	4.44 ± 7.55	1.47 ± 12.19	3.15 ± 7.98	0.53	0.44	0.68
Ewing score	0.72 ± 0.78	0.83 ± 0.84	0.91 ± 0.9	0.48 ± 0.56	0.006	0.69	0.008

*SFN, Small Fiber Neuropathy, SBP, Systolic Blood Pressure, DBP, Diastolic Blood Pressure. Bold values refer to statistically significant results*.

No significant difference was found between the “Definite SFN” and “No SFN” groups in terms of Q-Sweat, QST and CVT outcomes.

As regards skin biopsy, a significant difference in IENFD was highlighted between the “Definite SFN” and “No SFN” groups (4.6 ± 3.0 and 8.0 ± 2.4 fibers/mm, respectively; *P* < 0.0001).

ESC differed significantly between the “Definite SFN” and “No SFN” groups both in the hands (60.2 ± 16.7 vs. 75.0 ± 8.9 μS; *p* < 0.0001) and feet (70.2 ± 16.5 vs. 81.6 ± 7.0 μS; *P* < 0.0001).

Regarding LEP, only the amplitude of the N2P2 complex after foot stimulation differed between the 2 groups (14.0 ± 13.3, vs. 24.1 ± 12.3 μV, *P* < 0.0001).

### Correlations Between the Tests

The only significant inter-test correlation is between skin biopsy and ESC. Indeed, when the average foot ESC is related to the patient's weight (foot conductance/kg), this variable is correlated with the IENFD measured by skin biopsy (number of fiber/mm^3^) with a ρ coefficient of 0.38 (*P* < 0.0001). This correlation is stronger (ρ = 0.58, *P* < 0.0001) if the IENFD is also related to patient body weight. Conversely, there is no significant correlation between the IENFD and foot ESC if this variable is not related to body weight (ρ = 0.18, *P* = 0.005).

### Diagnostic Performance of the Investigations

The diagnostic performance of the investigations was evaluated by studying the normality or abnormality of each test in each patient according to the final diagnosis in patients with a definite diagnosis (i.e., in 104 patients with a “definite SFN” and 87 patients with “no SFN” with the exception of patients for whom the diagnosis was “possible SFN”). The results of the sensitivity (Se), specificity (Sp), positive predictive value (PPV) and negative predictive value (NPV) calculations are summarized in Table 4.

**Table 4:** Diagnostic performance of each test in terms of sensitivity, specificity, PPV (Positive Predictive Value) and NPV (Negative Predictive Value) which were determined from the final diagnosis of SFN based *per se* on the abnormality of two tests.

	**Total population**	**Definite SFN**	**Possible SFN**	**No SFN**	**Sensitivity**	**Specificity**	**PPV**	**NPV**
					**(CI 95%)**	**(CI 95%)**		
**Total**	245	102	53	90				
**Skin biopsy**								
Normal	155	42	33	80	0.58	0.91	0.88	0.66
Abnormal	84	59	17	8	(0.48–0.68)	(0.83–0.96)		
**QST**								
Normal	87	28	24	35	0.72	0.39	0.57	0.55
Abnormal	155	72	29	54	(0.62–0.81)	(0.29–0.50)		
**Q-Sweat**								
Normal	131	44	28	59	0.53	0.69	0.65	0.58
Abnormal	97	50	20	27	(0.43–0.64)	(0.58–0.78)		
**ESC study**								
Normal	145	40	26	79	0.60	0.89	0.86	0.66
Abnormal	97	61	26	10	(0.50–0.70)	(0.80–0.94)		
**LEP**								
Normal	135	32	32	71	0.66	0.89	0.88	0.68
Abnormal	86	63	15	8	(0.56–0.76)	(0.80–0.94)		
**CVT**								
Normal	214	84	45	85	0.15	0.96	0.81	0.50
Abnormal	27	15	8	4	(0.09–0.24)	(0.89–0.99)		

*SFN, Small Fiber Neuropathy; QST, Quantitative Sensory Testing; ESC, Electrochemical Skin Conductance; LEP, Laser Evoked Potentials; CVT, Cardio-Vascular Testing*.

The test with the best sensitivity in our study is the QST, with a Se of 72%, but a low Sp of 39%. The exploration with the best specificity is cardiovascular exploration, with a Sp of 96% but only 15% Se. Quantification of IENFD by skin biopsy has a Se of 58% and a Sp of 91%. LEP have a Se of 66%, a Sp of 89%. ESC measurement is characterized by a good Sp of 89% and a Se of 60%. The Q-Sweat has a Se of 69% and a Sp of 53%.

### Identification of a Diagnostic Strategy

Based on previous calculations, we concluded that the four most interesting tests were skin biopsy, LEP, ESC measurement, and QST. The calculations of Se, Sp, PPV, and NPV of the different combinations of these tests (with two-test abnormality as a criterion for diagnosis of SFN) are summarized in Table 5.

**Table 5:** Comparison of different diagnostic strategies for small fiber neuropathy.

**Tests combination**	**Total population**	**Definite SFN**	**Possible SFN**	**No SFN**	**Sensitivity (CI 95%)**	**Specificity (CI 95%)**	**PPV**	**NPV**
**Total**	245	102	53	90				
**Skin biopsy** **+** **ESC measurement** **+** **LEP** **+** **QST**								
<2 abnormal tests	108	8	21	79	0.92 (0.85–0.97)	0.88 (0.79–0.94)	0.9	0.91
≥2 abnormal tests	137	94	32	11				
**Skin biopsy** **+** **ESC measurement** **+** **LEP**								
<2 abnormal tests	138	28	28	82	0.67 (0.57–0.76)	1.00 (0.96–1.00)	1	0.73
≥2 abnormal tests	107	74	25	8				
**Skin biopsy** **+** **LEP** **+** **QST**								
<2 abnormal tests	149	29	36	84	0.72 (0.62–0.8)	0.93 (0.86–0.98)	0.92	0.75
≥2 abnormal tests	96	73	17	6				
**Skin biopsy** **+** **ESC measurement** **+** **QST**								
<2 abnormal tests	166	34	42	90	0.73 (0.61–0.81)	0.91 (0.83–0.96)	0.9	0.75
≥2 abnormal tests	79	68	11	0				
**LEP** **+** **ESC measurement** **+** **QST**								
<2 abnormal tests	137	27	28	82	0.74 (0.62–0.82)	0.91 (0.83–0.96)	0.9	0.76
≥2 abnormal tests	108	75	25	8				

*QST, Quantitative Sensory Testing; ESC, Electrochemical Skin Conductance; LEP, Laser Evoked Potentials; PPV, Positive Predictive Value; NPV, Negative Predictive Value*.

The best combination is the combination of the four tests (skin biopsy, LEP, ESC measurement, and QST) with a Se of 92%, a Sp of 88%, a PPV of 90% and a NPV of 91%. Among the three-test combinations, skin biopsy, ESC measurement and LEP generated a Se of 67% but a Sp and a PPV of 100% and a NPV of 73%.

## Discussion

### Diagnostic Strategy

Our results show that the best combination of tests to diagnose SFN comprises skin biopsy, LEP, ESC, and QST. The combination of these 4 tests generates a PPV of 90%, specificity of 88% with sensitivity of 92% and a NPV of 91%. This combination has the advantage of being based on sensitivity and specificity data calculated previously but also allows rational exploration of small fibers. Indeed, skin biopsy is a structural exploration whilst LEP and QST involve functional exploration of sensory aspects (A-delta and C fibers, respectively) and ESC explores the autonomic aspect. It should be noted that, of the four explorations, we propose LEP, QST, and ESC, which were the three tests recommended by Lefaucheur et al. (24) after comparing five diagnostic methods (not including skin biopsy or Q-Sweat) (24).

### Diagnostic Performance of Each Test

According to our results, QST is the most sensitive test for SFN diagnosis (72% Se, 40% Sp). This high sensitivity may be explained by the subjectivity of the test, which is based on patient self-reporting.

The second most sensitive exploration was LEP with a Se of 66% and a Sp of 90%. These results differ from the data published by Di Stefano et al. (78% Se, 81% Sp), (25) but the latter were obtained using skin biopsy as the gold standard.

The results generated with Q-Sweat were less interesting than those reported by Thaisetthawatkul et al. (23) 82% Se and 89% Sp vs. 64% Se and 54% Sp. This can be explained by differences in the number of studied sites (3 in our study (foot, proximal leg and forearm) instead of 4 (foot, distal leg, proximal leg and forearm) and in the interpretation of the results. Indeed, whereas we considered the test abnormal when the response was abnormal at one site, Thaisetthawatkul et al. used a composite criterion that required an abnormal value at two sites or at one site where a symptom was reported by the patient.

In our study, 59% Se and 88% Sp were recorded for the ESC measurement, which is similar to the results reported by Lefaucheur et al. (24).

Using the diagnosis based on the complete evaluation, as detailed above in the “final diagnosis” paragraph, skin biopsy has a Se of only 58% and a Sp of 92% in our study. This sensitivity is lower than the published data which range from 69 to 90% (3, 26, 27). However, these results were obtained in studies in which clinical examination was the only gold standard for SFN diagnosis. Our results also differ from those recently published by Devigili et al. which report a sensitivity of 94.3% and a specificity of 91.9% (28). It could be explained by methodological differences: we did not include clinical signs as a criteria for SFN but only used tests results in our population of patients with suggestive symptoms. This discrepancy suggests that patients with clinical signs are more likely to have an abnormal IEFND.

Therefore, it is uncertain whether skin biopsy can be considered as a reference method for the diagnosis of SFN. A significant proportion of the SFNs could be related to functional impairment of small fibers, detectable by functional explorations, but without fiber destruction as evidenced in biopsy. However, there could also be a difference in sensitivity between the various staining techniques used to read the slides in fiber counting. The technique used by Provitera et al. (29) with marking of the basal lamina ensures a more reliable fiber count and affects the sensitivity of the technique. A comparative study of the two techniques would be necessary to clarify this issue. The problem of uniformity of techniques and standards poses an additional difficulty in the interpretation of skin biopsy.

### Correlations

DN4 surprisingly did not differ between the groups “Definite SFN”, “no SFN” and “possible SFN.” This result confirms that although DN4 is a screening tool for SFN, a high DN4 score is not sufficient to assess the diagnosis of SFN.

Interestingly, no significant correlation was established between the raw exploratory data (including sensory and autonomic nervous system investigations). The only significant inter-test correlation in our study is a correlation between IENFD and skin conductance in the feet, which is significant only when the latter variable is related to patient weight. This result is consistent with the data reported by Novak (30).

### Causes

The distribution of underlying causes in patients with SFN in our cohort is consistent with some published data (22, 31). Indeed, the dominant causes are diabetes, glucose intolerance and Sjogren's syndrome as well as other dysimmune causes (the slightly higher prevalence of these causes in our cohort can be explained by the fact that some patients were recruited by the Internal Medicine Department). However, a more recent study in 2018 showed a high prevalence of sodium channelopathies by mutation of the SCN9A, SCN10A and SCN11A genes (which accounted for 16.8% of the causes found in 921 patients) (32). This result is not reflected in our cohort because the search for channelopathy was not systematically part of the SFN recommended aetiological assessment at that time.

In our study, etiological investigations were performed once the diagnosis of SFN was made. Concerning the medical background of our patients, in the “possible SFN” group, 4 patients had Sjogren's syndrome and 1 had diabetes; in the “no SFN” group, 7 patients had Sjogren's syndrome and 2 had diabetes; in the “definite SFN” group, 11 patients had Sjogren's syndrome, 12 had diabetes and 3 had an history of alcohol abuse or an history of cancer chemotherapy.

### Limits

One of the main methodological criticisms made in 2008 of Devigili's study (22) was the incorporation bias (the investigations assessed in terms of diagnostic value in the study were also included in the criteria used for the diagnosis of SFN). Botez and Herrmann pointed out that this bias was likely to overestimate the sensitivity of the tests (33). This incorporation bias is also present in our study. However, it is impossible to avoid it at the present time, given the absence of a real gold standard for the diagnosis of SFN.

Recently Devigili et al. questionned the relevance of resarching SFN in patients without suggestive clinical signs (28). We believe that including in our research patients with neurological symptoms only is interesting because it reflects the population in which SFN is suspected in clinical practice. In this population, it is intersting to notice that functional tests can be abnormal although biopsy do not show IEFD reduction.

The single center and retrospective nature of our study are other methodological limitations.

Finally, uncertainty remains for patients classified as “possible SFN” (when test results were borderline or when the test results could have been modified by a treatment or condition).

### Strengths

To date and to the best of our knowledge, our study is the first to compare a combination of 6 tests in the diagnosis of SFN.

Furthermore, the study population is similar to the population encountered in daily practice and the difficulties associated with SFN diagnosis, i.e., patients with suggestive symptoms (mainly pain) without extensive fiber neuropathy. A number of published studies investigated populations combining patients with pure SFN and mixed neuropathy of both small and large fibers (as indicated by abnormal nerve conduction). Our study avoids this pitfall by selecting only patients with normal nerve conduction.

Finally, we propose an original diagnostic approach based on our initial results, by calculating the PPV and NPV of different combinations of tests. As far as we are aware, this has not been done before since it requires a large patient cohort and varied diagnostic techniques.

## Conclusion

Our study shows the interest of skin biopsy, Laser Evoked Potentials, Quantitative Sensory Testing and Electrochemical Skin Conductance measurement for the diagnosis of Small Fiber Neuropathy. A combination of these four tests produces a PPV of 90% and a NPV of 91%. This test combination is likely to improve the diagnosis of SFN and must be investigated prospectively.

The role of new techniques (confocal corneal microscopy, new immunohistochemical methods for skin biopsy analysis, etc.), in the diagnosis of SFN, has yet to be established. This poses a challenge in the future.

## Data Availability Statement

The datasets generated for this study are available on request to the corresponding author.

## Ethics Statement

The studies involving human participants were reviewed and approved by Declaration to the national information science and liberties commission (CNIL) N°2150347. Written informed consent for participation was not required for this study in accordance with the national legislation and the institutional requirements.

## Author Contributions

VF: patients management, data collection, data analysis, paper writing, and submission. AG: study design, patients management, and proof reading. BA: patients management, data analysis, and proof reading. PC: patients management, data analysis, and proof reading. VR: data analysis. SE: pathologic examination of skin biopsies and proof reading. EU-C: pathologic examination of skin biopsies and proof reading. AP-L: study design, patients management, data analysis, and proof reading.

## Conflict of Interest

The authors declare that the research was conducted in the absence of any commercial or financial relationships that could be construed as a potential conflict of interest.

## References

[1] Gorson KC Herrmann DN Thiagarajan R Brannagan TH Chin RL Kinsella LJ. Non-length dependent small fibre neuropathy/ganglionopathy. J Neurol Neurosurg Psychiatry. (2008) 79:163–9. 10.1136/jnnp.2007.128801 17911181

[2] Lauria G Bakkers M Schmitz C Lombardi R Penza P Devigili G. Intraepidermal nerve fiber density at the distal leg: a worldwide normative reference study. J Peripher Nerv Syst. (2010). 15:202–7. 10.1111/j.1529-8027.2010.00271.x 21040142

[3] Lauria G Cornblath DR Johansson O McArthur JC Mellgren SI Nolano M. EFNS guidelines on the use of skin biopsy in the diagnosis of peripheral neuropathy. Eur J Neurol. (2005) 12:747–58. 10.1111/j.1468-1331.2005.01260.x 16190912

[4] McCarthy BG Hsieh S-T Stocks A Hauer P Macko C Cornblath DR. Cutaneous innervation in sensory neuropathies evaluation by skin biopsy. Neurology. (1995) 45:1848–55. 10.1212/WNL.45.10.1848 7477980

[5] Fruhstorfer H Lindblom U Schmidt WC. Method for quantitative estimation of thermal thresholds in patients. J Neurol Neurosurg Psychiatry. (1976) 39:1071–5. 10.1136/jnnp.39.11.1071 188989 PMC1083305

[6] Zaslansky R Yarnitsky D. Clinical applications of quantitative sensory testing (QST). J Neurol Sci. (1998) 153:215–38. 10.1016/S0022-510X(97)00293-1 9511880

[7] Low PA Caskey PE Tuck RR Fealey RD Dyck PJ. Quantitative sudomotor axon reflex test in normal and neuropathic subjects. Ann Neurol. (1983) 14:573–80. 10.1002/ana.410140513 6316835

[8] Low VA Sandroni P Fealey RD Low PA. Detection of small-fiber neuropathy by sudomotor testing. Muscle Nerve. (2006) 34:57–61. 10.1002/mus.20551 16718689

[9] Kakigi R Shibasaki H Tanaka K Ikeda T Oda K-I Endo C. CO2 laser-induced pain-related somatosensory evoked potentials in peripheral neuropathies: correlation between electrophysiological and histopathological findings. Muscle Nerve. (1991). 14:441–50. 10.1002/mus.880140510 1651448

[10] Lefaucheur J-P Debray S Jarry G. Laser evoked potentials using the Nd:YAG laser. Muscle Nerve. (2001) 24:496–501. 10.1002/mus.1032 11268021

[11] Treede R-D Lorenz J Baumgärtner U. Clinical usefulness of laser-evoked potentials. Neurophysiol Clin Neurophysiol. (2003) 33:303–14. 10.1016/j.neucli.2003.10.009 14678844

[12] Casellini CM Parson HK Richardson MS Nevoret ML Vinik AI. Sudoscan, a noninvasive tool for detecting diabetic small fiber neuropathy and autonomic dysfunction. Diabetes Technol Ther. (2013) 15:948–53. 10.1089/dia.2013.0129 23889506 PMC3817891

[13] Gordon Smith A Lessard M Reyna S Doudova M Robinson Singleton J. The diagnostic utility of Sudoscan for distal symmetric peripheral neuropathy. J Diabetes Complications. (2014) 28:511–6. 10.1016/j.jdiacomp.2014.02.013 24661818 PMC4219320

[14] Mayaudon H Miloche P-O Bauduceau B. A new simple method for assessing sudomotor function: relevance in type 2 diabetes. Diabetes Metab. (2010) 36:450–4. 10.1016/j.diabet.2010.05.004 20739207

[15] Tesfaye S Boulton AJM Dyck PJ Freeman R Horowitz M Kempler P. Diabetic Neuropathies: Update on Definitions, Diagnostic Criteria, Estimation of Severity, and Treatments. Diabetes Care. (2010) 33:2285–93. 10.2337/dc10-1303 20876709 PMC2945176

[16] Rolke R Magerl W Campbell KA Schalber C Caspari S Birklein F. Quantitative sensory testing: a comprehensive protocol for clinical trials. Eur J Pain. (2006) 10:77–88. 10.1016/j.ejpain.2005.02.003 16291301

[17] Rolke R Baron R Maier C Tolle TR Treede R- D Beyer A. Quantitative sensory testing in the German Research Network on Neuropathic Pain (DFNS): Standardized protocol and reference values. Pain. (2006) 123:231–43. 10.1016/j.pain.2006.01.041 16697110

[18] Novak P Quantitative autonomic testing. J Vis Exp. (2011) 53:2502 10.3791/2502 PMC3196175 21788940

[19] Devos D Creac'h C Laureau E Bourriez JL Guieu JD Les potentiels évoqués au laser thulium. Valeurs normatives aux membres supérieurs et inférieurs. Neurophysiol Clin Neurophysiol. (2000) 30:313–22. 10.1016/S0987-7053(00)00228-8 11126643

[20] Ewing DJ Martyn CN Young RJ Clarke BF. The value of cardiovascular autonomic function tests: 10 years experience in diabetes. Diabetes Care. (1985) 8:491–8. 10.2337/diacare.8.5.491 4053936

[21] Low PA Benarroch EE. Laboratory evaluation of autonomic function. LWW. (2008) 179–208.16106634

[22] Devigili G Tugnoli V Penza P Camozzi F Lombardi R Melli G. The diagnostic criteria for small fibre neuropathy: from symptoms to neuropathology. Brain. (2008) 131:1912–25. 10.1093/brain/awn093 18524793 PMC2442424

[23] Thaisetthawatkul P Fernandes Filho JAM Herrmann DN. Contribution of QSART to the diagnosis of small fiber neuropathy: QSART for small fiber neuropathy. Muscle Nerve. (2013) 48:883–8. 10.1002/mus.23891 23649502

[24] Lefaucheur J-P Wahab A Planté-Bordeneuve V Sène D Ménard-Lefaucheur I Rouie D. Diagnosis of small fiber neuropathy: a comparative study of five neurophysiological tests. Neurophysiol Clin Neurophysiol. (2015) 45:445–55. 10.1016/j.neucli.2015.09.012 26596193

[25] Di Stefano G La Cesa S Leone C Pepe A Galosi E Fiorelli M. Diagnostic accuracy of laser-evoked potentials in diabetic neuropathy. Pain. (2017) 158:1100–7. 10.1097/j.pain.0000000000000889 28267059

[26] Koskinen M Hietaharju A Kyläniemi M Peltola J Rantala I Udd B. A quantitative method for the assessment of intraepidermal nerve fibers in small–fiber neuropathy. J Neurol. (2005) 252:789–94. 10.1007/s00415-005-0743-x 15789134

[27] Lauria G Hsieh ST Johansson O Kennedy WR Leger JM Mellgren SI European federation of neurological societies/peripheral nerve society guideline on the use of skin biopsy in the diagnosis of small fiber neuropathy. Report of a joint task force of the European fe-deration of neurological Societies and the peripheral nerve society. Eur J Neurol. (2010) 17:903–e49. 10.1111/j.1468-1331.2010.03023.x 20642627

[28] Devigili G Rinaldo S Lombardi R Cazzato D Marchi M Salvi E. Diagnostic criteria for small fibre neuropathy in clinical practice and research. Brain. (2019) 142:3728–36. 10.1093/brain/awz333 31665231 PMC6906595

[29] Provitera V Gibbons CH Wendelschafer-Crabb G Donadio V Vitale DF Stancanelli A. A multi-center, multinational age- and gender-adjusted normative dataset for immunofluorescent intraepidermal nerve fiber density at the distal leg. Eur J Neurol. (2016) 23:333–8. 10.1111/ene.12842 26493160

[30] Novak P. Electrochemical skin conductance correlates with skin nerve fiber density. Front Aging Neurosci. (2016) 8:199. 10.3389/fnagi.2016.00199 27605912 PMC4995214

[31] Gemignani F Giovanelli M Vitetta F Santilli D Bellanova MF Brindani F. Non-length dependent small fiber neuropathy. A prospective case series. J Peripher Nerv Syst. (2010) 15:57–62. 10.1111/j.1529-8027.2010.00252.x 20433606

[32] de Greef BTA Hoeijmakers JGJ Gorissen-Brouwers CML Geerts M Faber CG Merkies ISJ. Associated conditions in small fiber neuropathy - a large cohort study and review of the literature. Eur J Neurol. (2018) 25:348–55. 10.1111/ene.13508 29112785 PMC5814938

[33] Botez SA Herrmann DN. Pitfalls of diagnostic criteria for small fiber neuropathy. Nat Clin Pract Neurol. (2008) 4:586–7. 10.1038/ncpneuro0920 18839004

